# Gait stability prediction through synthetic time-series and vision-based data

**DOI:** 10.3389/fspor.2025.1646146

**Published:** 2025-08-13

**Authors:** Mauricio C. Cordeiro, Ciaran O. Cathain, Vitor B. Nascimento, Thiago B. Rodrigues

**Affiliations:** ^1^ Department of Engineering & Informatics, Technological University of the Shannon, Athlone, Ireland; ^2^ Department of Sport & Health Sciences, Technological University of the Shannon, Athlone, Ireland; ^3^ SHE Research Centre, Technological University of the Shannon, Athlone, Ireland; ^4^ Department of Sport Science and Nutrition, Faculty of Science and Engineering, Maynooth University, Maynooth, Ireland; ^5^ Department of Physical Education, Pontifícia Universidade Católica do Paraná, Curitiba, Brazil

**Keywords:** synthetic data, gait stability, computer vision, SHAP values, MediaPipe pose estimation

## Abstract

**Introduction:**

Gait stability assessment in older adults is challenged by limited data availability and measurement complexity, particularly among vulnerable populations and in limited resource settings. We address three research questions: (1) can synthetic data accurately replicate the statistical properties of gait parameters in older adults? (2) how effectively do synthetic data-trained models predict the Margin of Stability (MoS) when tested on real-world data? and (3) what specific biomechanical features contribute most significantly to the MoS predictions in older adults? To address these challenges, the present study proposes a novel approach to gait stability prediction by integrating computer vision with a data-centric synthetic data generation (SDG) approach using accessible, low-cost technology.

**Methods:**

Using a public dataset from 14 healthy older adults (86.7 ± 6.2 years), we implemented a constraint-based SDG methodology that preserved biomechanical relationships through SDG metadata configuration and rank correlation-based constraints. Gait analysis was performed through a smartphone (Motorola Moto G5 Play) and the open-source MediaPipe algorithm to extract body landmarks from frontal plane gait videos, making the approach suitable for resource-limited settings.

**Results:**

Our approach achieved exceptional fidelity (97.09% overall) and maintained biomechanical variable relationships. The model trained exclusively on synthetic data (TSTR) outperformed the model trained on real data (TRTR), with error reductions (RMSE decreased by 56.3%, MAE by 58.2%, and MSE by 80.9%) and improved variance explanation (*R*^2^ increase of 31.2%). SHAP analysis revealed that the synthetic data approach enhanced feature attribution alignment with established principles, particularly for step width, BMI, and fall history.

**Discussion:**

Therefore, our results show that: (1) synthetic data accurately replicated gait parameters with high fidelity; (2) synthetic data-trained models outperformed real data-trained models in MoS prediction; and (3) step width, BMI, and fall history were the most significant predictors of MoS in older adults. These findings demonstrate the potential of synthetic biomechanical time series to overcome data scarcity, improve predictive modeling capabilities, and enhance clinical gait assessment through accessible, low-cost computer vision methods.

## 1 Introduction

Human walking is a method of locomotion involving the use of the two legs alternately to provide support and propulsion by at least one foot throughout the gait cycle (1). Consequently, gait is an individual trait in healthy subjects that can be used for personal identification (2). However, it changes with age (3, 4) and can be transformed by emotions (5), exercise-related or cognitive fatigue (6), or environmental factors (7). Assessing gait stability is particularly important in older adults, where impairments in walking can affect both independence and quality of life. Gait stability, pragmatically defined as the ability to walk without falling despite perturbations (8), is essential for maintaining active living. Therefore, various methods have been developed to assess gait stability, given that neuromuscular conditions and physical impairments can compromise balance control and lead to increased fall risk (9).

The consequences of mobility loss are severe for older adults. By age 70, approximately one-third report mobility restrictions, increasing to the majority by age 80. These limitations are linked to age-related declines in muscle strength, oxygen consumption, and sensory function, which collectively impair balance control and increase the risk of instability and falls (10–12). Early identification of gait abnormalities and effective quantification of stability in many clinical populations has gained significant interest as increased knowledge of balance deficits or compensatory strategies may aid rehabilitation and inform therapeutic interventions to improve quality of life and functional capacity (11).

As described in (13), gait stability assessment relies on biomechanical principles investigated at the center of mass (CoM), the weighted average of a body's mass. During walking, stability depends on two factors: (1) the position of the CoM relative to the base of support (BoS), which determines whether the body is within stable limits, and (2) CoM velocity, which creates momentum that must be controlled through corrective forces to maintain balance. The extrapolated center of mass (XCoM) extends the CoM concept by incorporating its velocity scaled by a person-specific constant, enabling stability predictions during motion. This stability analysis can be quantified using the margin of stability (MoS), defined as the signed distance between the XCoM and the BoS (13). This MoS serves as the prediction target for our machine-learning models.

However, gait-related assessment in older adults can face relevant challenges, including limited data availability, measurement complexity, and resource constraints due to a diverse set of problems, such as mobility constraints, cognitive impairments, variability in functional capacity, inconsistent adherence to assessment protocols, and the heterogeneity of age-related gait patterns (10–12). The integration of synthetic data generation (SDG) techniques has emerged as a promising approach to improve the accuracy and robustness of gait pattern modeling (14, 15). Researchers can address data scarcity, privacy concerns, and data quality challenges by generating synthetic data that replicates real-world statistical properties, enabling the training of more accurate machine learning models (16–19). Traditional synthetic data approaches emphasize fidelity, ensuring that synthetic data statistically resembles real-world data through distribution matching. Nevertheless, this singular focus might be insufficient for biomechanical applications because synthetic data with specific quality deficiencies can reduce predictive performance and distort model selection processes, compromising research integrity (20–24). Despite these challenges, there remains limited research on applying synthetic data generation to gait stability-related parameters, particularly those captured using computer vision-based methods. This research gap is significant given the SDGs’ potential benefits for augmenting gait datasets, improving model generalization across diverse populations and walking conditions, enabling more prediction-based stability assessment tools, and uncovering valuable information hidden within biomechanical data.

Based on the identified research gaps in applying SDG to gait stability, this study addresses three research questions: (1) Can synthetic data accurately replicate the statistical properties of gait parameters in older adults? (2) How effectively do synthetic data-trained models predict MoS when tested on real-world data? (3) What specific biomechanical features contribute most significantly to the MoS predictions in older adults? We hypothesise that synthetic data generated with biomechanical constraints will enhance machine learning model performance for MoS prediction in older adults beyond that achievable with real-world data alone.

We adopt a dual-evaluation approach, assessing both statistical resemblance and utility, which represents a necessary shift beyond conventional statistical metrics (such as Maximum Mean Discrepancy or Kullback-Leibler divergence) towards a comprehensive data-centric approach (20). In this context, data utility measures how effectively synthetic data enhances downstream applications when validated against real-world data, particularly regarding model generalization and predictive accuracy. This dual-metric evaluation framework ensures that synthetic data serves two important functions: (1) representing the statistical properties of original data, and (2) providing practical utility through comparative performance metrics when models trained on synthetic data are evaluated against real-world data (TSTR paradigm). By adopting a data-centric perspective, our SDG process aims to maintain biomechanical validity while addressing challenges in gait analysis, including limited sample sizes, inter-subject variability, and requirements for model generalization across the older adult population. Thereby, the synthesizer aims to generate sequential data (Centre of Mass position, CoM velocity, Margin of Stability) and static attributes (Age, Body Mass Index, Fall incidence history), with all synthetic samples undergoing comprehensive quality assessment to ensure biomechanical plausibility and effective dataset augmentation.

In summary, this paper assesses gait stability using metrics calculated from body landmark tracking via computer vision (25). We apply this approach to frontal plane video footage of healthy older adults (aged >65 years) during self-paced walking, employing synthetic data generation to enhance model training. For prediction, we employ Extreme Gradient Boosting (XGBoost) (26), selected for its capability to handle biomechanical data with complex, non-linear relationships. Model interpretability is enhanced through Shapley Additive Explanations (SHAP) values, which elucidate the contribution of each feature to the MoS predictions (27).

## 2 Methods

### 2.1 Data source and participants

**Figure 1:**
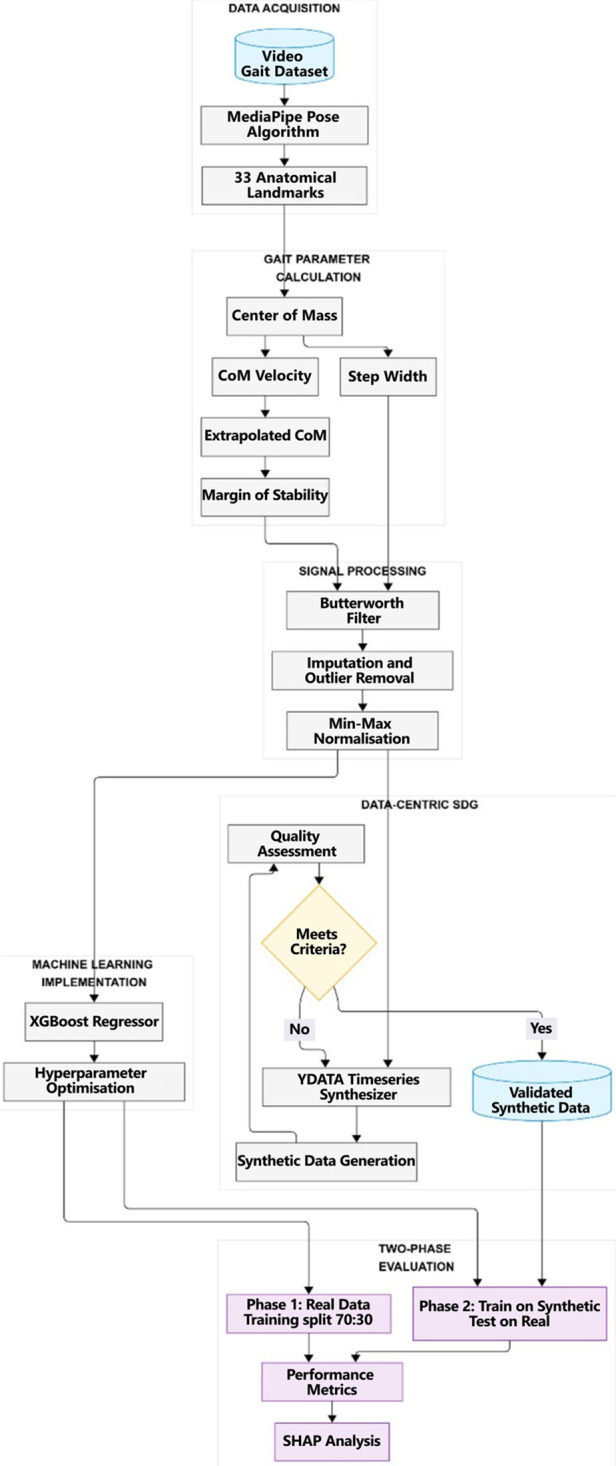
The flowchart of our methodology.

In data collection, we used a publicly available dataset (28) that was subsequently processed using the MediaPipe algorithm (see Figure 1). The dataset comprised 14 healthy older adults residing in a retirement home (11 female, 3 male participants). The participants’ mean (±standard deviation) age, height, and mass were 86.7 ± 6.2 years, 165.6 ± 9.9 cm, and 64.0 ± 12.5 kg, respectively. Each participant performed a standardized walking protocol, moving back and forth for one minute along a flat, 13 m pathway within a large room.

The walking sessions were recorded using two Motorola Moto G5 Play cell phones (Motorola, Chicago, IL), each with a 13-megapixel rear camera capturing high-definition 1080p video at 30 frames per second. Frontal plane recordings (positioned at 111 centimeters height, designated as “bottom” in the file naming convention) were used for gait analysis.

Accompanying the frontal plane video recordings, the dataset included participant metadata with demographic information and clinical test scores. Based on that, we acknowledge that gait analysis commonly requires multi-view perspectives or 3D motion capture systems. However, we selected frontal plane analysis as it enables assessment of medio-lateral stability parameters relevant for this specific evaluation in older adults and aligns with our focus on low-cost computer vision methods in resource-constrained clinical settings.

Despite the limited sample size (*n* = 14), this dataset is suitable for our research because it provides standardized gait data from a homogeneous population of older adults collected using a smartphone camera in a controlled setting. This smartphone-based recording facilitates reliable biomechanical measurements whilst addressing realistic challenges of data scarcity and equipment limitations commonly encountered in clinical practice.

#### 2.1.1 Computer vision and the MediaPipe pose approach

With the advancement of computer vision techniques, markerless gait analysis has become possible through video footage using pose estimation models such as BlazePose (29). These techniques involve using computer vision and machine learning (ML) algorithms to extract human poses and track the movement of the body's anatomical landmarks in 2D or 3D spaces over time.

**Figure 2:**
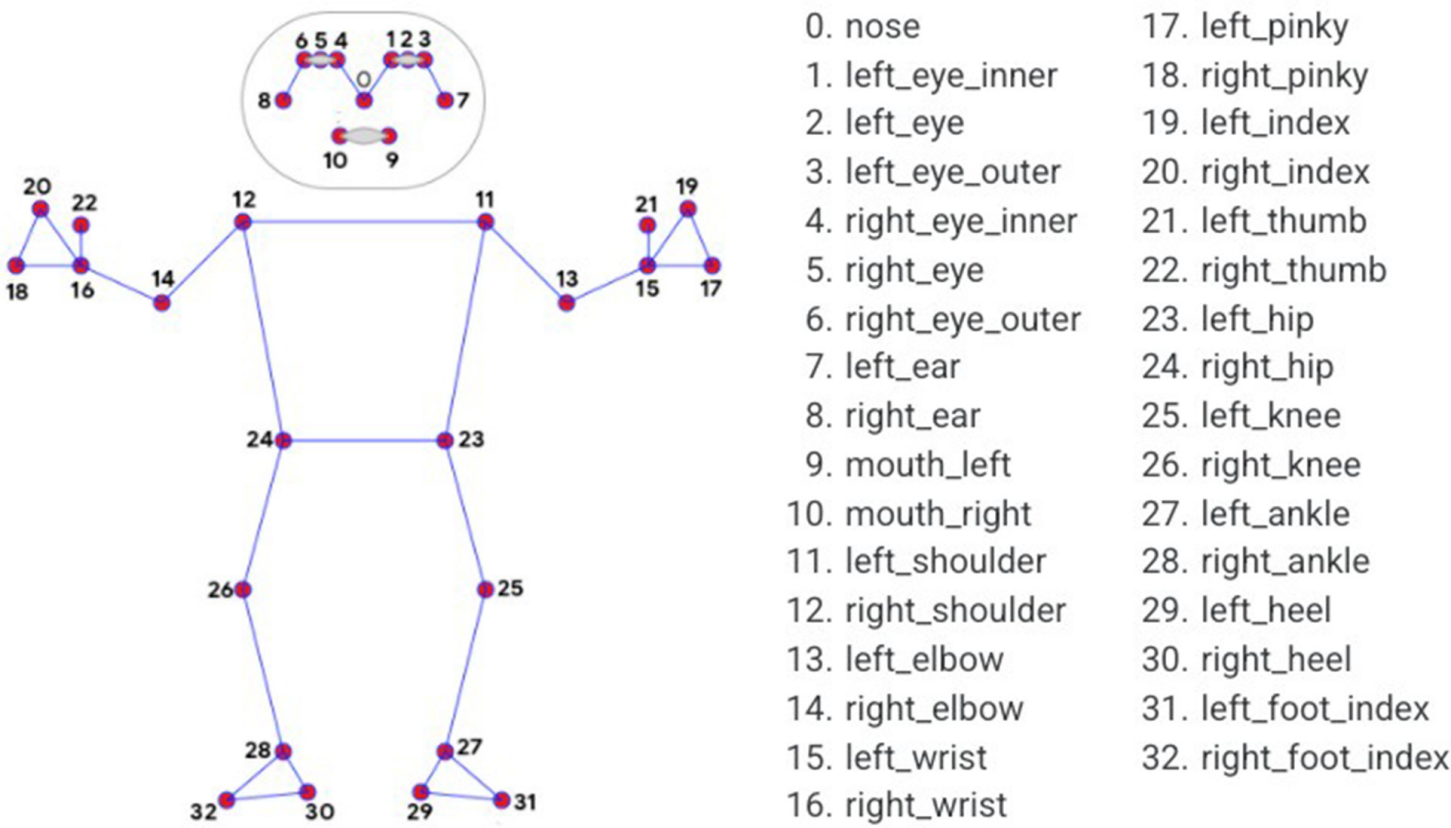
Mediapipe pose landmarks (59).

MediaPipe Pose (MPP), an open-source, cross-platform framework provided by Google, captures 2D human joint coordinates in each image frame, consisting of three pre-trained detection models: EfficientDet-Lite0, EfficientDet-Lite2, and the Single Shot Detector (SSD) MobileNetV2 Model trained on the COCO image dataset (30). MPP uses BlazePose (29), a lightweight ML architecture that performs quickly on mobile phones and PCs with CPU inference, extracting thirty-three 2D landmarks on the human body, as shown in Figure 2. Moreover, studies have demonstrated MediaPipe's feasibility against gold-standard motion capture systems for the measurement of angular variation for biomechanical evaluation (31), and for tracking gait parameters in the frontal plane with low mean absolute error (0.04–0.18 s) (32). Figure 2 illustrates the full set of MediaPipe Pose landmarks available for analysis. We used specific subsets of these landmarks for our gait stability calculations, as detailed in Table 1.

**Table 1:** Landmark coordinates required for kinematic measurements.

Calculation purpose	Landmarks used	Landmark numbers
Center of Mass (CoM)	Nose, Left/Right eyes (inner & outer), Shoulders, Hips, Elbows, Wrists, Knees, Ankles	0, 1, 2, 3, 4, 11, 12, 13, 14, 15, 16, 23, 24, 25, 26, 27, 28
Extrapolated Center of Mass (XCoM)	Left and Right Hips	23, 24
Step Width	Left and Right Ankles	27, 28

Several metrics were derived from the integration of MPP and the dataset itself, as outlined in Table 2. However, before that, to accurately calculate stability metrics such as the MoS, we included each participant's height and weight directly within the *MediaPipe* processing code for each video sequence, allowing the algorithm to generate stability metrics that account for individual anthropometric differences in COM displacement calculations across participants.

**Table 2:** Gait metrics and their origin and application.

Metric	Origin and application
COM^a^	We calculated the COM for each frame based on key body landmarks. Segmental COM values were weighted according to their proportional contribution to the total body weight, and a combined COM was computed.
Velocity of COM	By analyzing the COM's movement between frames, we calculated the velocity based on the frame acquisition rate, a crucial input for stability-related measures.
XCoM^a^	We computed using COM velocity and position data to provide an adjusted measure of stability for each frame.
MoS^a^	We determined it by calculating the distance between XCoM and the base of support, formed by the left and right ankles, for each frame. This quantifies stability dynamically as the participant moves.
Step Width	By analyzing ankle positions, we calculated the step width for each frame, which provides information about gait stability and variability.

^a^ COM, center of mass; XCoM, extrapolated center of mass; MoS, margin of stability.

As described in (13), one limitation in the estimation of MoS is the assumption of instantaneous adjustments in the center of pressure (CoP). CoP adjustments are constrained by the finite reaction time required for muscle activation. While this limitation has been explored in standing balance, it still needs to be investigated for walking dynamics. Although our *Mediapipe's* pose extraction works to capture gait dynamics, it has not fully addressed this previous limitation.

These are the formulas used to calculate these metrics. First, to calculate the COM for body segments, we used Equation 1:

(1) $$\begin{array}{c} \mathrm{CO}M_{\mathrm{segment}}=\frac{1}{n}\overset{n}{\underset{i=1}{\sum }}\;p_{i} \end{array}$$

Where $p_{i}$ represented the position vector of the *i* -th landmark within the segment (with x and y coordinates from the 2D video plane), and *n* is the total number of landmarks in the segment. While MediaPipe provided estimated z-coordinates, our analysis used the frontal plane (x,y) coordinates for consistency with our 2D video capture methodology.

The COM for the entire body is then calculated as a weighted sum of each segment's COM using anthropometric segment weight coefficients that represent each body segment's proportional mass relative to total body weight (Equation 2). These coefficients were: head (8.1%), torso (49.7%), arms (2.65% each), and legs (16.1% each), based on established anthropometry (33).

(2) $$\begin{array}{c} \mathrm{CO}M_{\mathrm{body}}=\frac{\sum_{\;j=1}^{m}\;w_{j}\cdot \mathrm{CO}M_{j}}{\sum_{\;j=1}^{m}\;w_{j}} \end{array}$$

Where $\mathrm{CO}M_{j}$ is the COM of the *j* -th body segment, $\;w_{j}$ is the weight coefficient of that segment as a proportion of total body weight, and *m* is the total number of body segments considered.

The velocity of the CoM is derived from the difference between successive CoM positions over time (Equation 3):

(3) $$\begin{array}{c} v_{\mathrm{COM}}(t)=f\cdot [\mathrm{CO}M_{\mathrm{body}}(t)-\mathrm{CO}M_{\mathrm{body}}(t-1)] \end{array}$$

Where *f* is the sampling frequency in frames per second and *t* represents the frame index.

Additionally, the XCoM is calculated as an extrapolation of the CoM based on its velocity, helping to determine stability (Equation 4):

(4) $$\begin{array}{c} \mathrm{XCoM}=\mathrm{CO}M_{\mathrm{body}}+\frac{v_{\mathrm{COM}}}{\omega_{0}} \end{array}$$

Where $\omega_{0}=\sqrt{\frac{g}{l}}$ is the eigenfrequency of the inverted pendulum, with $g=9.81\;m/s^{2}$ as gravitational acceleration and *l* l as the leg length. In our implementation, the leg length is computed as a fraction of the distance between the CoM and the midpoint of the ankles.

Moreover, we measured the MoS by evaluating the distances of the XCoM and CoM from the edge of the support base formed by the feet (Equation 5):

(5) $$\begin{array}{c} \mathrm{MoS}=\min(d_{\mathrm{XCoM}},d_{\mathrm{COM}}) \end{array}$$

Where $d_{\mathrm{XCoM}}$ and $d_{\mathrm{COM}}$ represent the perpendicular distances from XCoM and CoM to the boundary of the base of support, calculated using Equations 6, 7:

(6) $$\begin{array}{c} d_{\mathrm{XCoM}}=\frac{\left\|v_{\mathrm{XCoM}}\times v_{\mathrm{boundary}}\right\|}{\left\|v_{\mathrm{boundary}}\right\|} \end{array}$$

(7) $$\begin{array}{c} d_{\mathrm{COM}}=\frac{\left\|v_{\mathrm{COM}}\times v_{\mathrm{boundary}}\right\|}{\left\|v_{\mathrm{boundary}}\right\|} \end{array}$$

Finally, the step width between the left and right ankles is computed (Equation 8):

(8) $$\begin{array}{c} \mathrm{StepWidth}=\|r_{\mathrm{lef}t_{xy}}-r_{\mathrm{righ}t_{xy}}\| \end{array}$$

Where $r_{\mathrm{lef}t_{xy}}$ and $r_{\mathrm{righ}t_{xy}}$ represent the left and right ankle joint centers’ two-dimensional projections onto the frontal (xy) plane. Based on these tools, integrating datasets with MediaPipe's data extraction capabilities enabled us to capture metrics across the individuals’ walking sequences.

### 2.2 Data preprocessing

Data preprocessing is the subsequent step to ensure the quality and reliability of the analysis. This was conducted using Python's Pandas (version 2.2) (34) and Scipy (version 1.13.1) (35) libraries following a structured sequence of operations as detailed below.

To reduce the noise and variability in the raw data, a fourth-order Butterworth low-pass filter was applied (36). The filter's cutoff frequency was set to 4 Hz, commonly used in gait analysis to retain relevant gait dynamics while attenuating high-frequency noise (36). This filtering step smoothed the data and aimed to improve the accuracy of subsequent calculations.

We applied this filter independently to each landmark's x and y coordinates, ensuring zero-phase distortion and minimal signal delay. After filtering, the gait parameters (CoM, Step Width, MoS) were recalculated using the filtered landmark positions. This ensured that all subsequent analysis was based on refined trajectory data.

During video processing, the pose estimation algorithm can occasionally lose track of participants, particularly during rapid movements. These occasions result in missing data points for specific frames. To address this challenge, a mean imputation technique was employed using information from the previous and subsequent frames within the same walking sequence, maintaining the continuity of the movement trajectory. Also, we ensured that the data collection did not provide a number of missing values extremely high, i.e., greater than 20% of the total number of frames in one gait cycle, as suggested by (37).

We detected and removed outliers to maintain the integrity of the dataset. We implemented a statistical approach based on each variable's mean and standard deviation. First, the mean (μ) and standard deviation (σ) were calculated for each sequential parameter. A data point was classified as an outlier if it fell outside the interval μ ± k·σ, where k is a threshold factor. We selected k = 2 for our analysis, identifying values more than two standard deviations from the mean as outliers. This approach is a standard data preprocessing technique widely used in signal processing and anomaly detection (38). The two-standard-deviation threshold retains approximately 95% of the data, preserving the majority of valid observations and eliminating extreme values that could distort subsequent analyses.

The final preprocessing step involved normalizing the input variables introduced into the machine learning model on the TRTR (Training on real data, testing on real data) process. On TSTR (Training on synthetic data, testing on real data), we had one more of the same normalization approach after the SDG implementation, focusing on applying the normalized data to the Machine Learning (ML) model. Thereby, we applied *Min*–*Max* scaling, transforming each feature to a range between 0 and 1. This normalization ensures that features with different units and ranges contribute equally to the model without bias toward variables with larger numerical scales (39).

### 2.3 Synthetic data generation (SDG)

This research employed a data-centric approach to SDG for biomechanically-based time series data. The methodology centered on preserving specific biomechanical relationships while generating statistically representative synthetic samples. We employed YData's TimeSeriesSynthesizer [v2.0.0, *ydata-sdk Python* package (40)] to generate biomechanically constrained synthetic time-series data, configured via hierarchical metadata and correlation-preserving constraints (Table 3).

**Table 3:** Metadata configuration for biomechanical-based time series data.

Metadata component	Information provided to synthesizer	Utilization in synthesis process
Temporal structure	The sequential organization of biomechanical parameters across time points (Timestamp range: 0.0–7.2594)	Time-series architectures that preserve autocorrelation structure and temporal contingencies
Feature correlations	Multivariate relationship matrix (e.g., Step Width-MoS correlation: 0.768)	Multivariate sampling with covariance preservation; rank correlation-based approaches for maintaining interdependencies
Distributional parameters	Statistical moments of features, including skewness values	Normalizing flows; transformation functions that accurately model tail behaviors and central tendencies
Categorical frequencies	Cardinality and class distributions (14 unique participant IDs)	Stratified generation processes; conditional sampling with proper class balancing mechanisms
Variables boundaries	Domain constraints for biomechanical variables (e.g., Step Width: 0.002–0.266 m)	Constrained optimization; bounded generative functions with domain-appropriate activation mechanisms
Rank correlation-based biomechanical constraint	Rank-based correlation thresholds (Step Width-MoS: *ρ* > 0.7, Velocity-MoS: *ρ* < −0.3) derived from empirical biomechanical relationships	Distribution-invariant enforcement of biomechanical principles through rank correlation validation; preservation of stability-support relationships whilst accommodating individual variability

In the generation process, we incorporated a hierarchical architecture that respected temporal dependencies (stride-to-stride transitions) and entity-specific patterns (participant-level characteristics). Timestamp values were designated as the sequential sorting key, whilst participant identifiers were established as entity boundaries, enabling the synthesizer to capture both within-subject variability and between-subject differences in gait parameters.

We integrated domain-specific biomechanical constraints derived from established principles of locomotor stability. These constraints were designed to maintain relationships between gait parameters, particularly the positive correlation between step width and margin of stability (reflecting increased base of support) (41) and the inverse relationship between the center of mass velocity and margin of stability (reflecting reduced control at higher speeds) (42, 43).

#### 2.3.1 SDG technique

We used the TimeSeriesSynthesizer framework and implemented additional rank correlation-based dependency constraints to enforce biomechanical relationships in the generated data. The model architecture incorporated the following key components.

##### 2.3.1.1 Metadata configuration

The synthesizer was initialized with time series metadata specifying Timestamp as the temporal sorting key and ID as the entity identifier. This configuration established appropriate boundaries for learning temporal dynamics whilst preserving participant-specific characteristics.

##### 2.3.1.2 Rank correlation-based constraint implementation

We developed custom constraint functions that evaluated relationships between biomechanical variables through rank statistics. Specifically, we utilized Spearman rank correlations to enforce relationships between key biomechanical parameters whilst maintaining their individual distributional properties. This approach enabled us to:

(1) Enforce a positive correlation between step width and margin of stability (*ρ* > 0.7, reflecting the observed value of 0.768 in the real data correlation matrix).
(2) Maintain the inverse relationship between the center of mass velocity and margin of stability (*ρ* < −0.3, capturing the observed value of −0.487 in the real data correlation matrix).

These correlation thresholds were implemented via custom validation functions that verified whether generated data maintained the specified biomechanical relationships. The functions calculated Spearman rank correlations between the relevant variables and validated that they met the predetermined thresholds derived from established biomechanical principles.

##### 2.3.1.3 Variables boundary preservation

The synthesizer maintained the derived domains for biomechanical variables, including each variable's minimum and maximum values, such as step width (0.002–0.266 m) and margin of stability (0.004–0.054 m).

##### 2.3.1.4 Model training and data generation

Finally, we trained the synthesizer using 1,878 timesteps of original data from 14 participants. This constrained model subsequently generated the synthetic dataset with equivalent dimensionality, preserving distributional characteristics and variable relationships.

### 2.4 SDG quality process and its assessment

To evaluate the quality of the synthetic data, we implemented an assessment framework addressing data fidelity and utility. This process comprised two components: (1) synthetic data fidelity evaluation and (2) predictive performance assessment through a supervised machine learning model.

#### 2.4.1 Synthetic data fidelity metrics

Synthetic data fidelity was assessed through complementary statistical approaches. The *Python* packages used are *SDV (version 1.18.0)* (44), *SDMetrics (version 0.17.0)* (45), and *Scipy (version 1.13.1)* (35).

##### 2.4.1.1 Univariate distribution similarity

We employed the Kolmogorov–Smirnov (46) complement (KSComplement) for continuous variables and the Total Variation complement (TVComplement) for categorical variables. These metrics quantified distribution similarity on a scale from 0 to 1, with higher values indicating greater fidelity (45). Column shapes were evaluated with an aggregate score across all variables.

##### 2.4.1.2 Bivariate relationship preservation

We assessed pairwise variable relationships through correlation pattern analysis, quantifying the degree to which the synthetic data maintained the interrelationships in the original dataset (45).

##### 2.4.1.3 Hellinger distance calculation

We computed Hellinger distances between original and synthetic distributions for each variable and overall. This probabilistic divergence measure (scaled between 0 and 1, with lower values indicating greater similarity) assessed distribution similarity sensitive to location and shape differences (47).

#### 2.4.2 Machine learning approach

To evaluate the practical utility of the synthetic data, we implemented a supervised machine learning framework using a gradient-boosting regression model trained to predict the margin of stability from gait parameters and participants’ characteristics.

##### 2.4.2.1 Model architecture

We employed XGBoost regression models, a decision tree-based ensemble ML technique, selected for their capacity to capture non-linear relationships in biomechanical data across various studies (48–50). The XGBoost minimizes the models’ residuals and increases the predictive power by combining weak learners (51). Using XGBoost, we aimed to identify the relationships between biomechanical parameters and the Margin of Stability, thereby establishing a model capable of accurately estimating stability from gait variables.

##### 2.4.2.2 Hyperparameter optimization

To maximize predictive performance, we implemented a comprehensive grid search strategy using *sci-kit-learn's GridSearchCV* combined with 5-fold cross-validation. This approach evaluated all possible combinations of the following hyperparameter values for the XGBoost model (Table 4). The grid search process evaluated 135 different hyperparameter combinations (3 × 3 × 3 × 2 × 2), with each combination subjected to 5-fold cross-validation. The optimal configuration was selected to minimize the Mean Absolute Error (MAE) across validation folds.

**Table 4:** Hyperparameter optimization.

Classifier	Hyperparameter	Optimized parameter values
Extreme gradient boosting (XGBoost)	n_estimators	[100, 200, 300]
	max_depth	[3, 5, 7]
	learning_rate	[0.01, 0.1, 0.2]
	subsample	[0.8, 1.0]
	colsample_bytree	[0.8, 1.0]

##### 2.4.2.3 Comparative evaluation

Our machine learning implementation followed a two-phase approach to evaluate predictive performance and the utility of our SDG.

(1) *Phase 1* — We trained the model on real-world gait data using a 70:30 train-test split of the original dataset, so we tested it on the held-out real data. We called it the TRTR (Training on Real, Testing on Real) approach.
(2) *Phase 2* — We trained the model exclusively on synthetic data and then evaluated its performance by testing on the complete real-world dataset. We called it the TSTR (Training on Synthetic, Testing it on Real) approach.

This design allowed us to directly assess whether models trained on synthetic data could generalize effectively to real-world observations. We employed XGBoost for both phases to predict the Margin of Stability variable using the input variables listed in Table 5.

**Table 5:** Input and output variables for MoS prediction.

Metric	Units	Type	Description
X Coordinate CoM^a^	Meters (m)	Input (Numeric)	X-coordinate of the Center of Mass
Y Coordinate CoM^a^	Meters (m)	Input (Numeric)	Y-coordinate of the Center of Mass
Step width	Meters (m)	Input (Numeric)	Width of the step during gait
CoM_Velocity	m/s	Input (Numeric)	Velocity of the Center of Mass
Age	Category	Input (Categorical)	0: Middle-old (75–84), 1: Oldest-old (≥85)
BMI^a^	Category	Input (Categorical)	0: Underweight (<23), 1: Healthy weight (23–30)
Fall incidence in the last 6 months (Fall History)	Category	Input (Categorical)	No Falls (0), At Least One Fall (1)
MoS	Meters (m)	Output (Numeric)	Margin of Stability

^a^ COM, center of mass; BMI, body mass index.

In terms of performance assessment, we evaluated model performance using a comprehensive set of metrics to assess prediction accuracy for the MoS output variable (Table 5).

*Mean Absolute Error (MAE)*. Measures the average absolute difference between predicted values (${\hat{y}}_{i}$) and actual values ($y_{i}$) across all *n* observations, often used to deal with the problem of differentiability (see Equation 9). The lower the value, the better the result. A value of zero indicates a perfect fit.

(9) $$\begin{array}{c} MAE=\frac{1}{n}\overset{n}{\underset{i=1}{\sum }}\;|y_{i}-{\hat{y}}_{i}| \end{array}$$

*Mean Squared Error (MSE)*. Quantifies prediction error by calculating the average squared differences between predicted values and actual observations (see Equation 10). It is used to overcome the problem of differentiability in MAE. The lower the value, the better the result. A value of zero indicates a perfect fit.

(10) $$\begin{array}{c} MSE=\frac{1}{n}\overset{n}{\underset{i=1}{\sum }}\;{\left(y_{i}-{\hat{y}}_{i}\right)}^{2} \end{array}$$

*Root Mean Squared Error (RMSE)*. Provides a measure of the average magnitude of prediction errors in the same units as the target variable, facilitating interpretation (see Equation 11). RMSE is more sensitive to outliers than MAE, but its expression in the original unit of measurement makes it important for this biomechanical application.

(11) $$\begin{array}{c} RMSE=\sqrt{\frac{1}{n}\overset{n}{\underset{i=1}{\sum }}\;{\left(y_{i}-{\hat{y}}_{i}\right)}^{2}}=\sqrt{MSE} \end{array}$$

*Coefficient of Determination (R² score)*. Indicates the proportion of variance in the target variable that is predictable from the independent variables. Its values range from 0 to 1, indicating no fit and fit. The higher the value, the better the result, which means the closer the R2 value is to 1, the better the model is fitted. *R*^2^ is calculated as 1 minus the ratio of the sum of squared errors (SSE) to the total sum of squares (SST), where $\bar{y}$ represents the mean of the observed values (see Equation 12).

(12) $$\begin{array}{c} R^{2}=1-\frac{\sum_{i=1}^{n}\;{\left(y_{i}-{\hat{y}}_{i}\right)}^{2}}{\sum_{i=1}^{n}\;{\left(y_{i}-\bar{y}\right)}^{2}}=1-\frac{SSE}{SST} \end{array}$$

These metrics allowed for a thorough comparison between our two phases, providing insight into the utility of synthetic data for MoS prediction. Additionally, they are widely recognized as reliable measures for evaluating gait parameter predictions (52, 53).

##### 2.4.2.4 Model interpretability

To understand feature contributions and enhance model transparency, we implemented Shapley Additive Explanations (SHAP) (27). For our XGBoost model, we used the SHAP Tree Explainer, which efficiently calculates contribution values for each feature input.

The SHAP analysis identified the biomechanical variables with the greatest influence on MoS predictions and enabled comparisons of feature importance patterns between models trained on real or synthetic data. This interpretability framework was essential for validating that synthetic data preserved the relationships present in the original dataset and respected well-established gait parameters principles, especially for older adults’ gait. However, this model interpretability tool can face challenges, including computational intensity for large datasets and sensitivity to noise (54).

## 3 Results

The following subsections detail the results of the experiments conducted.

### 3.1 Synthetic data fidelity analysis

#### 3.1.1 Overall fidelity metrics

The synthetic data demonstrated exceptional fidelity across multiple evaluation dimensions.

These metrics indicate that the metadata parameters used in this approach produced synthetic data that closely mirrors the statistical properties of the original dataset while maintaining these variables’ relationships (Table 6).

**Table 6:** Overall synthetic data fidelity assessment.

Evaluation metric	Score	Interpretation
Column shapes	98.51%	Near-perfect preservation of univariate distributions
Column pair trends	95.67%	Excellent maintenance of bivariate relationships
Overall fidelity	97.09%	Very high overall synthetic data quality

#### 3.1.2 Distribution similarity analysis

The Kolmogorov–Smirnov complement (KSComplement) and Total Variation complement (TVComplement) scores for individual variables demonstrated exceptional preservation of univariate distributions (Table 7).

**Table 7:** Distribution similarity metrics by variable.

Variable	Metric	Score
CoM X coordinate	KSComplement	0.978
CoM Y coordinate	KSComplement	0.977
Step Width	KSComplement	0.978
Margin of stability	KSComplement	0.979
Timestamp	KSComplement	1.00
Age	TVComplement	0.990
Fall history	TVComplement	1.00
BMI	TVComplement	0.986
CoM velocity	KSComplement	0.974

**Figure 3:**
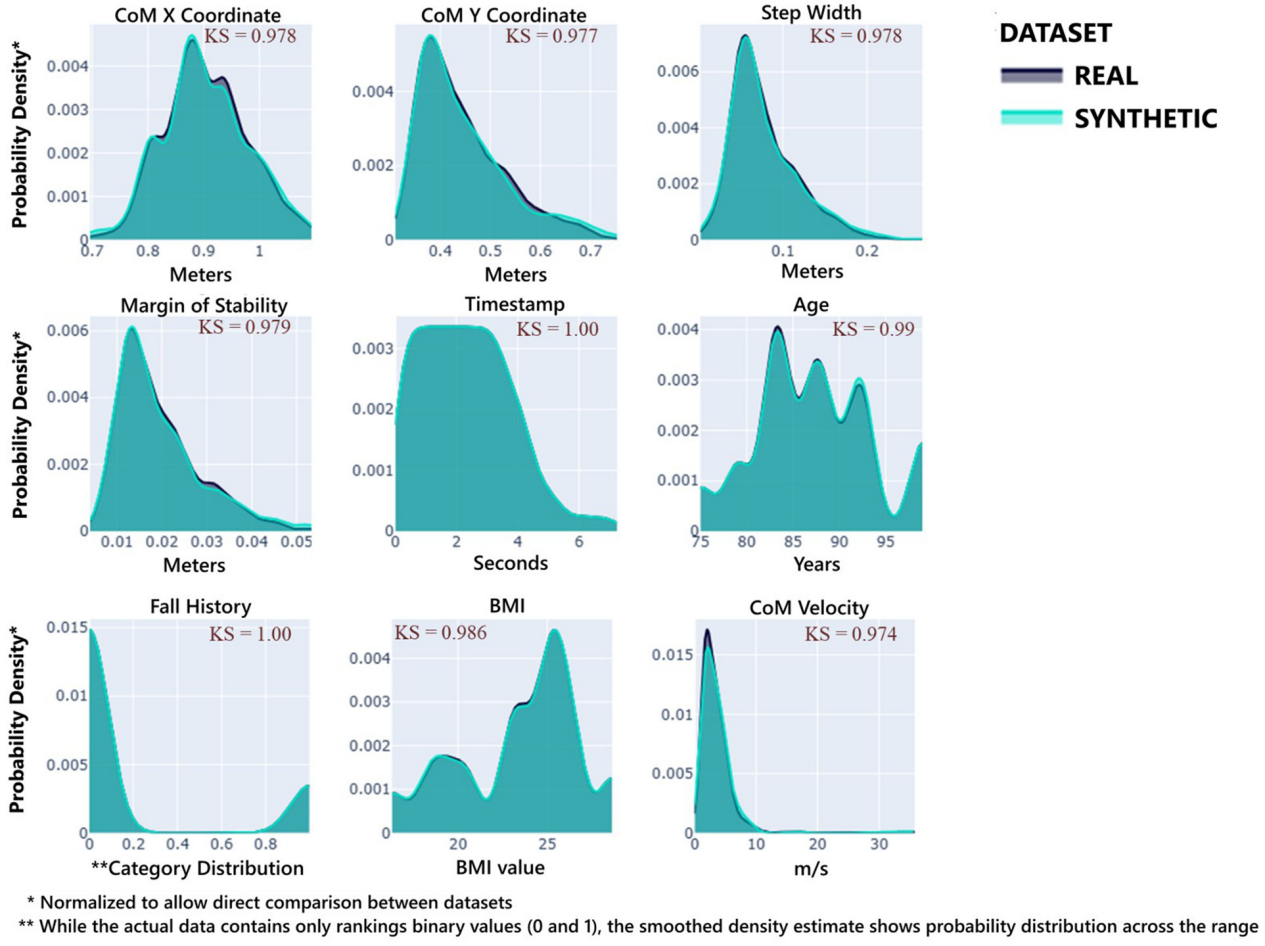
Distribution comparison between original and synthetic data. Each subplot shows its data distribution, synthetic data (blue color), and real data (gray color). Additionally, the KS Complement is introduced at the top right of each subplot to demonstrate each variable's fidelity performance.

All variables exhibited scores above 0.97, with categorical variables (e.g., Fall History) achieving perfect preservation (1.0). This demonstrates that the synthetic data maintained the distributional characteristics of all gait parameters, which is visually confirmed in Figure 3, with a high degree of overlap between the real and synthetic distributions.

#### 3.1.3 Probabilistic divergence assessment

The overall Hellinger Distance (HD) = 0.0193 indicated excellent alignment between the original and synthetic distributions. Specifically, the biomechanical variables at the core of our rank correlation-based constraints — Step Width (HD = 0.0333), MoS (HD = 0.0332), and CoM Velocity (HD = 0.0447), retained low divergence despite the constraint enforcement.

### 3.2 Performance metrics comparison

The synthetic data-based model (TSTR) demonstrated superior predictive performance compared to the real-data-only approach, with error metrics reduced by 56%–81% and variance explanation (*R*^2^) improved by 31.2% (Table 8). This finding suggested that this approach may attenuate random variability and preserve its variable relationships, enhancing the model's ability to capture patterns in these gait parameters.

**Table 8:** Performance comparison between the model performance on “TRTR and TSTR-based” approaches.

Performance metric	TRTR^a^	TSTR^a^	*Δ*—improvement
Mean Absolute Error (MAE)	3.4672	1.4479	−2.0193 (58.2%)
Mean Squared Error (MSE)	18.7308	3.5790	−15.1518 (80.9%)
Root Mean Squared Error (RMSE)	4.3279	1.8918	−2.4361 (56.3%)
*R*^2^ Score	0.7321	0.9603	+0.2282 (31.2%)

^a^ TRTR, training on real data and testing on real data; TSTR, training on synthetic data and testing on real data.

**Figure 4:**
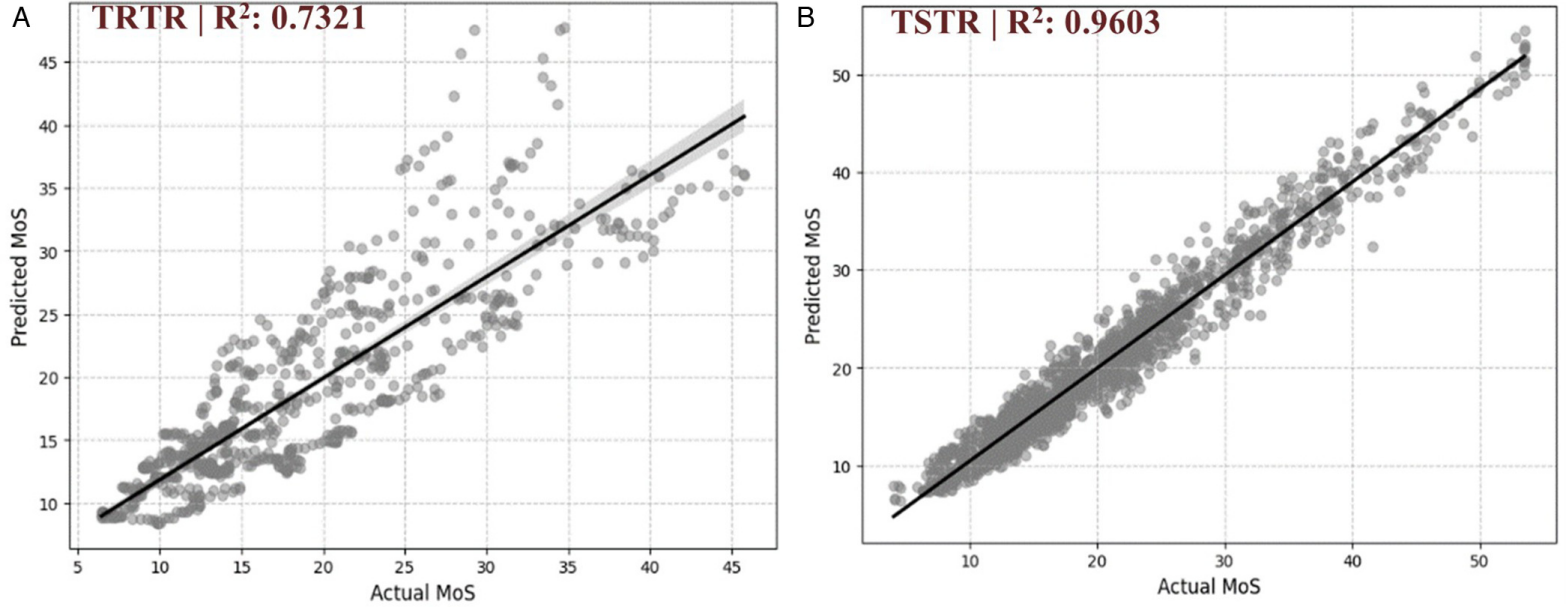
Comparison of predicted vs. actual Margin of Stability (MoS) values. **(A)** Model trained on real data and tested on real data (TRTR), with an *R*^2^ score of 0.7321, indicating the proportion of variance in the actual MoS values explained by the model predictions. **(B)** Model trained on synthetic data and tested on real data (TSTR), with an *R*^2^ score of 0.9603.

Figure 4 displays the TRTR and TSTR models’ actual vs. predicted MoS values. For the TRTR model [Figure 4A], while the model captured the general trend (*R*^2^ = 0.7321), variance is evident through the widespread scatter around the regression line. Prediction accuracy appeared limited at higher MoS values (>30), with a dispersion of predictions. In contrast, the TSTR model [Figure 4B] improved the prediction accuracy. The tighter clustering of points around the regression line visually confirmed the superior performance metrics (*R*^2^ = 0.9603). Prediction accuracy remained consistent across the entire range of MoS values, including at higher magnitudes where the TRTR model showed limitations.

### 3.3 Feature attribution analysis

The SHAP (Shapley Additive Explanations) value analysis revealed redistributions of feature influence when using synthetic data. SHAP values quantified the contribution of each feature to model predictions, with higher values indicating a greater impact on the outcome. For example, as shown in Table 9, the Step Width Mean SHAP value increased by 128.3%, better reflecting its established role in stability control. The “Fall History” increased the mean SHAP value (1789.3%), suggesting enhanced sensitivity to this risk factor. The BMI increased the mean SHAP value (175.7%). Finally, the CoM_Velocity decreased mean SHAP value (37.6%), consistent with the inverse relationship with stability. This last one potentially happened because of the rank correlation-based constraint enforcement.

**Table 9:** Mean SHAP values comparison between models.

Feature	TRTR	TSTR	Directional change
Y_Coordinate_CoM	5.720624	5.469393	↓ (4.4%)
X_Coordinate_CoM	0.733808	0.673718	↓ (8.2%)
Step_Width	0.719645	1.642897	↑ (128.3%)
BMI	0.587171	1.618933	↑ (175.7%)
Age	0.479669	0.724091	↑ (50.9%)
CoM_Velocity	0.289063	0.180252	↓ (37.6%)
Fall history	0.026910	0.508422	↑ (1,789.3%)

These shifts in SHAP values indicated that this synthetic data approach realigned model feature attribution to better reflect established gait principles, especially for the older adult population.

**Figure 5:**
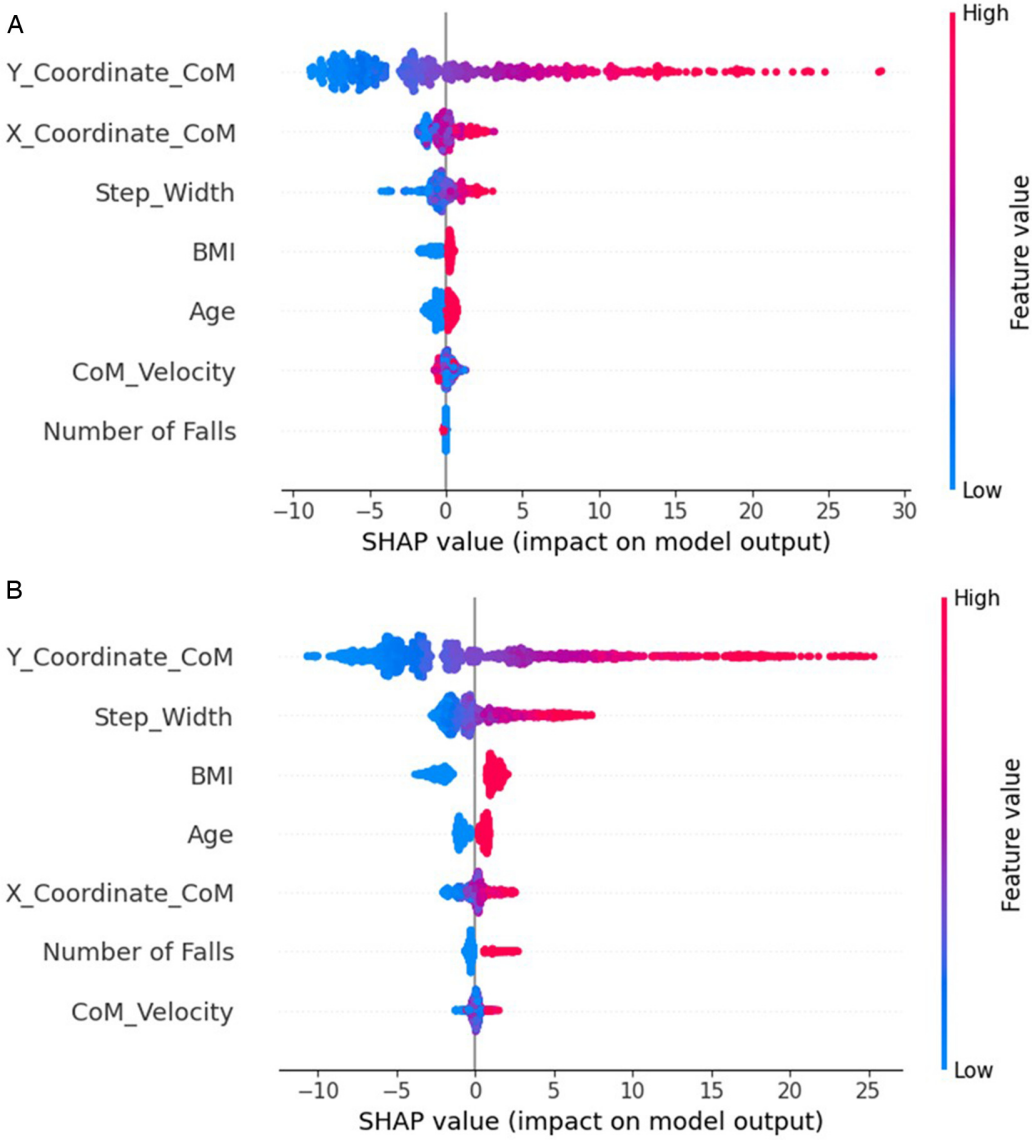
SHAP summary plot for TRTR **(A)** and TSTR **(B)** models. The color gradient reflects feature values, where red indicates higher feature values and blue indicates lower feature values.

Figure 5 demonstrates the feature attribution patterns for the TRTR and TSTR models. For TRTR [Figure 5A], the horizontal distribution of points represented the SHAP value impact on model output, while color indicated feature value magnitude (blue for lower values, red for higher values). The *Y_Coordinate_CoM* showed the most influence on predictions, with positive and negative contributions depending on the value. *Step_Width* demonstrated a modest impact, particularly compared to the CoM-coordinates-based parameters. In contrast, Figure 5B reveals the altered feature attribution pattern in the TSTR model. *Step_Width* gained greater impact in the predictions, rising to second position in importance. As mentioned before, this realignment better reflected established biomechanical principles regarding the role of step width in stability control. Fall History also showed enhanced contribution compared to the TRTR model, indicating improved sensitivity to risk factors.

## 4 Discussion

This study investigated the application of SDG to gait stability prediction in older adults, addressing three research questions about data fidelity and utility, predictive performance, and model interpretability. Our findings demonstrated that this synthetic data approach accurately replicates data properties of gait parameters and can also enhance predictive modeling capabilities beyond what is achievable with real-world data alone in older adults.

The high fidelity metrics, column shapes at 98.51%, column pair trends at 95.67%, and overall fidelity at 97.09%, and subsequently preservation of variable distributions (above 0.97 in KSComplement and TVComplement scores) addressed an important challenge in movement science, maintaining biomechanical data validity whilst augmenting a limited dataset. For example, preserving categorical variables related to fall history (TVComplement = 1.00) is essential for clinical risk assessment applications. The low Hellinger distance (0.019) further confirmed the similarity between the original and synthetic distributions. Therefore, these findings demonstrated that our data-centric approach successfully captured univariate distributions and bivariate relationships. These fidelity metrics align with similar methodological approaches using evaluation metrics proposed by the Synthetic Data Vault (SDV) framework and/or Hellinger distance across all variables (15, 55). Importantly (15), used SDV-based metrics, such as KSComplement, to assess the data fidelity of synthetic gait data generated for multiple sclerosis patients and demonstrated strong performance results with most metrics over 0.75. These findings advance beyond traditional statistical distribution matching approaches by demonstrating that constraint-based synthetic data can effectively embody relationships between domain-specific biomechanical variables. The implementation of rank correlation-based constraints to maintain established relationships between step width and margin of stability (*ρ* > 0.7) and between CoM velocity and stability margins (*ρ* < −0.3) represents a methodological solution in biomechanically-based SDG, depending on each research goal.

Regarding the data utility, the most significant finding is that models trained exclusively on synthetic data (TSTR) demonstrated superior predictive performance compared to models trained on real data alone (TRTR). The reductions in error metrics (MAE by 58.2%, MSE by 80.9%, and RMSE by 56.3%) and improved variance explanation (*R*^2^ increase of 31.2%) highlight the potential of the metadata configuration process used in this SDG application. These results align with the guidance from (19) for an SDG data-centric framework, emphasizing that high-quality synthetic data should achieve statistical fidelity and enhance utility in downstream tasks.

The predictive performance in Figure 4 further supports this interpretation, showing that while the TRTR model struggles with prediction accuracy at higher MoS values (>30 mm), the TSTR model maintains consistent accuracy across the entire range. This suggests that our SDG approach improved prediction accuracy at the boundaries of stability values, thus enhancing, with caution, reliability in assessing marginal dynamic stability profiles. For instance, an 85-year-old patient recovering from a mobility-constrained procedure with MoS values around 25–35 mm (where the TRTR model showed poor accuracy) could support a decision-making process that led to an unnecessarily prolonged rehabilitation procedure. The TSTR model's enhanced performance in this MoS range enables better identification of patients at the threshold of safe mobility, supporting responsible decision-making for this healthcare example.

The SHAP analysis revealed redistributions of feature influence in the TSTR model, with notable increases in the importance of Step Width (128.3%), BMI (175.7%), and Fall History (1,789.3%). These changes suggest that the synthetic data generation process influenced the model's feature attribution patterns. Our findings relate to (56) research that, via a VICON motion capture system in 105 healthy individuals (52.87 ± 19.09 years), demonstrated that step width (part of a ‘base of support’ domain, as identified by the factor analysis, which included step width and step time) was a significant predictor of medio-lateral margin of stability, explaining 26% of the variance (*p* < 0.0001). Their equipment setup differs from our low-cost computer vision footage-capturing approach, which makes our application emerge as an alternative for a wider clinical accessibility solution.

Therefore, this SHAP analysis reinforces two biomechanical principles: (1) step width serves as an active control mechanism for lateral stability, and (2) clinical history (previous falls) and anthropometric factors (BMI) influence gait stability strategies. Regarding a predictive-based perspective, CoM velocity demonstrated relatively low importance in both models (TRTR and TSTR), which appears contrary to established biomechanical theory regarding the inverse relationship between velocity and stability margins, which (8, 57) demonstrated their relations that higher velocities can reduce the time available for corrective responses. This contrary relation to our findings can be explained by the experimental context of self-selected speeds. When participants walk at their preferred speed, they could optimize their gait pattern for stability and comfort, effectively minimizing the destabilizing effects of velocity that would be more apparent under imposed speed conditions. At self-selected speeds, older adults could adopt conservative velocity strategies that maintain their stability within comfortable margins, reducing the variance in velocity-stability relationships that machine learning models potentially rely on to detect feature importance. Therefore, this underscores the importance of considering the experimental context when interpreting SHAP values in gait modeling.

Our findings have significant implications for gait assessment in similar older adult populations. The SDG addresses persistent challenges, including limited sample sizes and constraints in extensive data collection from vulnerable populations. The improved predictive performance of the TSTR model suggests that synthetic data augmentation could enhance the accuracy of fall risk assessment tools based on stability metrics, particularly for the oldest-old population (≥85 years), where fall risk assessment carries the highest urgency (58), which aligns with the data that one-third of 70-year-olds and most 80-year-olds report mobility restrictions, which involves physical losses, including decreases in limb maximum muscle force and power (10, 12).

### 4.1 Limitations

Despite the promising results, some limitations exist: First, the sample size of 14 older adults we used, while comparable to many gait studies, remains relatively small for comprehensive synthetic data validation for this population. Future research should assess the scalability of a similar approach with more diverse cohorts representing different age groups, pathological conditions, and environmental contexts. Following this future perspective, for pathological populations (e.g., Parkinson's disease, stroke survivors), this SDG approach would require condition-specific constraint development. For instance, Parkinson's patients would potentially exhibit reduced step width variability and altered center of mass control, which would necessitate modified gait parameters correlation thresholds and potentially additional constraints reflecting disease-specific compensatory strategies. The SDG would need to preserve these pathological gait patterns whilst maintaining biomechanical plausibility.

Additionally, the dataset's composition (11 female, 3 male participants) reflects a gender imbalance that may limit the generalizability of our synthetic data generation approach. Future investigations should examine whether synthetic data generation maintains gender-specific biomechanical relationships and consider implementing gender-balanced original datasets for synthesis, or alternatively, generate synthetic data specifically for minority gender classes to address representation gaps. In other words, implement gender as a stratification variable in the metadata configuration, enabling the synthesizer to generate synthetic samples that maintain gender-specific proportions and biomechanical characteristics for synthetic data quality across gender groups.

Second, our SDG focused on frontal plane stability parameters derived from a single walking condition (level walking at self-paced speed). Thus, future work should extend this methodology to sagittal plane stability and responses to perturbations, which represent important aspects of stability control not captured in the current analysis. The SDG metadata configuration framework could accommodate frontal and sagittal plane parameters simultaneously, enabling the generation of synthetic data that preserves biomechanical relationships in both planes (e.g., coordination between medio-lateral and anterior-posterior stability strategies).

Third, while the MediaPipe algorithm provided acceptable pose tracking, advanced pose estimation algorithms optimized for biomechanical gait analysis could improve stability metrics’ precision and data quality. Advanced algorithms with improved joint tracking precision and enhanced robustness to occlusion could reduce measurement noise in center of mass calculations and step width detection, directly improving the accuracy of margin of stability computations.

### 4.2 Future clinical validation process

Our study demonstrates predictive-based SDG implementation in gait stability assessment, but comprehensive clinical validation is essential before widespread decision-making implementation. Critical validation steps include multi-site clinical trials across diverse healthcare settings to ensure methodology robustness, systematic testing in pathological populations (e.g., stroke survivors, Parkinson's disease patients, balance disorder patients) to validate synthetic data accuracy for clinical gait patterns, and longitudinal studies tracking patients over time to assess balance disorders prediction accuracy and rehabilitation monitoring capabilities. Additionally, clinical workflow integration requires practitioner usability studies, clinical decision support validation, and environmental robustness testing across different lighting conditions, clothes, and clinical environments to ensure smartphone-based pose tracking maintains accuracy in real-world settings.

Moreover, cost-effectiveness analysis comparing the methodology against current standard-of-care approaches, along with clinical outcome studies demonstrating improved patient outcomes, reduced falls, and enhanced quality of life, will be essential for healthcare adoption. Our current findings, relevant for stability assessment, provide an initial exploration for these validation efforts and position this methodology as a promising tool for low-cost gait stability assessment in clinical practice.

## 5 Conclusion

This study demonstrates that SDG with specific biomechanically based constraints can accurately replicate gait stability parameters in older adults, addressing our three research questions regarding data fidelity, utility, and interpretability. First, our approach replicated gait parameter statistical properties with exceptional fidelity: column shapes (98.51%), column pair trends (95.67%), and overall fidelity (97.09%), with variable distributions exceeding 0.97 in KSComplement and TVComplement scores and low Hellinger distance (0.019) confirming excellent alignment between original and synthetic distributions. Second, synthetic data-trained models (TSTR) demonstrated superior predictive performance compared to real data-trained models (TRTR), achieving substantial error reductions (MAE by 58.2%, MSE by 80.9%, RMSE by 56.3%) and improved variance explanation (*R*^2^ increase of 31.2%), whilst maintaining consistent accuracy across the entire MoS range, including at boundary stability values (>30 mm) where TRTR models showed limitations. Third, SHAP analysis revealed step width, BMI, and fall history as the most significant MoS predictors, with the synthetic data approach enhancing feature attribution alignment with established biomechanical principles through increased importance of step width (128.3%), fall history (1,789.3%), and BMI (175.7%). The redistribution of feature importance in the TSTR model revealed this approach's strength: amplifying signals aligned with established gait stability principles, creating a more interpretable predictive framework. This approach, combined with accessible computer vision methodology, contributed to advancing gait stability assessment with implications for fall risk monitoring. By enabling accurate stability assessment through smartphone cameras rather than expensive motion capture systems, this methodology could help in scenarios where resources and mobility are limited for fall risk screening, enabling earlier interventions that improve decision-making from clinicians and physiotherapists, especially through similar explainable machine learning implementation. Moreover, the improved accuracy at boundary stability values supports precision-based gait stability assessment for the most vulnerable patients regarding the margin of stability. Future work should extend these SDG-data-driven methods to diverse populations and stability conditions, potentially developing new predictive-based stability assessment solutions with varied goals, including clinical settings.

## Data Availability

Publicly available datasets were analyzed in this study. This data can be found here: the original and synthetic data generated in this study can be found at: https://github.com/mauriciomau0/Gait-Stability-Prediction-Through-Synthetic-Time-Series-and-Vision-Based-Data. Additionally, the synthetic dataset was generated from a publicly available dataset that can be accessed here: https://doi.org/10.6084/m9.figshare.c.5515953.v1.
